# Health-related quality of life in Switzerland: normative data for the SF-36v2 questionnaire

**DOI:** 10.1007/s11136-019-02161-5

**Published:** 2019-03-08

**Authors:** Katharina Roser, Luzius Mader, Julia Baenziger, Grit Sommer, Claudia E. Kuehni, Gisela Michel

**Affiliations:** 1 Department of Health Sciences and Health Policy, University of Lucerne, Frohburgstrasse 3, PO Box 4466, 6002 Lucerne, Switzerland; 2 Swiss Childhood Cancer Registry, Institute of Social and Preventive Medicine, University of Bern, Bern, Switzerland; 3 Department of Paediatrics, University Children’s Hospital, University of Bern, Bern, Switzerland

**Keywords:** Health-related quality of life, SF-36, SF-36v2, Population norm, Switzerland, Weighting coefficients, Patient-reported outcome (PRO), Patient-reported outcome measure (PROM)

## Abstract

**Purpose:**

Health-related quality of life (HRQOL) is an important concept to describe well-being of the general population and persons with diseases. The short form-36 (SF-36) is a widely used questionnaire assessing self-reported HRQOL in eight health domains. The aims of this study were to provide normative data for the SF-36 version 2 (SF-36v2) for all language regions in Switzerland and weighting coefficients to calculate two summary measures for physical and mental health.

**Methods:**

A random representative (regarding age, sex, and language region) sample of people living in Switzerland aged 18–75 years in 2015 was eligible for our questionnaire survey. We calculated the eight health domain subscales for different subsamples based on sociodemographic characteristics. Two summary measures for physical and mental health were derived using data-based factor score coefficients and calculated for the subsamples.

**Results:**

A total of 1209 persons completed the SF-36v2 (mean age 48.7 years, 58.1% women). The SF-36v2 was valid and reliable in Switzerland. Physical health was better in men (*p* = 0.012) and younger persons (*p* < 0.001). Mental health was better in men (*p* < 0.001) and older persons (*p* < 0.001). Regarding regional differences, we found better physical (*p* = 0.002) and mental (*p* < 0.001) health in German speaking persons compared to French and Italian speaking persons.

**Conclusions:**

This paper presents the first SF-36v2 normative data for Switzerland, which are based on a recent study in a representative sample. Our normative data and weighting coefficients will enable future studies to compare HRQOL assessed by the SF-36 in healthy and diseased persons to a representative Swiss sample.

**Electronic supplementary material:**

The online version of this article (10.1007/s11136-019-02161-5) contains supplementary material, which is available to authorized users.

## Background

Health-related quality of life (HRQOL) is an important concept to describe subjective well-being of the general population and persons suffering from a disease. HRQOL is an important patient-reported outcome (PRO). To evaluate HRQOL, it is important to consider the persons’ views and experiences, and the multidimensional nature of well-being [1]. PROs might differ from assessments by health care professionals and objective indicators of health. It is therefore important to take patients’ perspectives of well-being into account [2, 3]. HRQOL is influenced by health status, but depends also on characteristics such as gender, age, migration background, level of education, employment status, and type of employment [4–13]. These characteristics differ between populations; consequently, HRQOL differs also. Moreover, HRQOL might change over time [13]. Thus, population-specific and up-to-date data on HRQOL are needed.

Based on those considerations, the medical outcomes study short form-36 (SF-36) was developed and became one of the most widely used patient-reported outcome measure (PROM) assessing HRQOL [1]. The SF-36 assesses HRQOL using eight subscales to measure two components of HRQOL: physical and mental health [14]. It can be completed in 5–10 min and has a high acceptability and data quality [15]. Valid normative data derived from a well-defined and representative sample of the general population are essential to be able to interpret results from specific groups such as particular patient populations [16]. So far, no normative data for the SF-36 questionnaire was available for Switzerland. The only Swiss-based validation of the SF-36 questionnaire has been a French language version applied in 1992 to a sample of young adults (SF-36v1, *n* = 1007, mean age 30 years, 53% women) living in the French speaking part of Switzerland [17]. This study is outdated, and results apply only to the French speaking region of Switzerland. Thus, many Swiss studies have compared their results to normative data from other countries restraining the value of these comparisons since countries differ in socio–economic and cultural characteristics [18] known to be related to HRQOL [19–22].

To overcome this gap, we collected representative and up-to-date data on of the general population of Switzerland including all language regions using the SF-36 version 2 (SF-36v2). These can serve as comparison data for future studies investigating HRQOL in Swiss people. Specifically, we aimed to provide (1) normative data for the eight health domain subscales of the SF-36v2 and (2) weighting coefficients for the construction of physical and mental HRQOL summary measures, and normative data for the summary measures.

## Methods

### Study sample

The random and representative sample of the general population of Switzerland was obtained from the Swiss Federal Statistical Office (SFSO) [23], drawn according to the distributions of age, sex, and language region (German, French, Italian) in Switzerland. It included 3000 households (2153 households from the German part, 711 from the French part, and 136 from the Italian part of Switzerland) of Swiss residents, in which at least one person was aged 18–75 years on 31 December 2014. The sample included 7052 persons in total. We included persons aged 18–75 years in 2015 (*n* = 5644) in our survey.

### Procedure

We contacted eligible persons individually with an information letter in one of the national languages of Switzerland (German, French, or Italian) as indicated by the SFSO. Approximately 2 weeks later, they received the questionnaire with a cover letter and a pre-paid return envelope unless they refused. Non-respondents received a reminder letter with an additional copy of the questionnaire and another pre-paid return envelope. Data were collected between May 2015 and June 2016.

### SF-36 questionnaire

We used validated versions of the SF-36v2 questionnaire [24] in German, French, and Italian. Translations of the SF-36 were shown to be culturally appropriate and comparable [25, 26]. The SF-36v2 questionnaire consists of 36 items. All but one item are assigned to one of the eight health domains covering various aspects of physical and mental health: physical functioning (PF, 10 items), physical role functioning (RP, 4 items), bodily pain (BP, 2 items), general health perceptions (GH, 5 items), vitality, (VT, 4 items), social role functioning (SF, 2 items), emotional role functioning (RE, 3 items), and mental health (MH, 5 items) [14]. Health domain subscales consist of the sum scores of the assigned items. Out of the eight subscales, each representing one health domain, two summary measures can be constructed: the physical component summary (PCS) for self-perceived physical health and the mental component summary (MCS) for self-perceived mental health. To construct the summary measures, scores of the eight health domain subscales are weighted according to their contributions to the two summary measures and summed up [24, 27].

### Covariates

To compare participants and non-participants, sex and age of participants and non-participants were derived from the SFSO data. Age was categorized into six categories (18–25, 26–35, 36–45, 46–55, 56–65, ≥ 66 years). Participants were considered having a migration background if they were not born in Switzerland (SFSO data), not Swiss citizen (SFSO data), or not Swiss citizen since birth (questionnaire data). The highest achieved education was assessed in the questionnaire and classified into four categories (compulsory schooling (corresponding to International Standard Classification of Education (ISCED) 1–2), vocational training (ISCED 3–4), upper secondary education (ISCED 5), and university education (ISCED 6–8)) [28, 29]. Employment status (employed, unemployed, retired), living in a partnership (yes, no), civil status (single, married, divorced/widowed), children living in the household (no, yes), and the presence of a chronic condition or health problem (no, yes) were assessed in the questionnaire. Different subsamples were defined based on these covariates. Additional subsamples were defined for the three language regions in Switzerland (German, French, Italian) based on the questionnaire language.

### Statistical analysis

The SF-36v2 data were cleaned according to the manual of the SF-36v2 [24, 27]. Subscale raw scores of the eight health domain subscales were converted into percentage scores (referred to as *p* scores), i.e. scores were standardized with 0 representing the lower and 100 representing the upper bound of the scale. Higher scores indicate better HRQOL. Subscale raw scores were imputed if at least half of the subscale items were available using the mean value of the available items of the respective subscale [24]. We conducted sensitivity analyses to compare results using imputed and non-imputed health domain subscale scores.

We examined the representativeness of our study sample by comparing it to the sample of non-participants using the following available covariates: sex, age, nationality (Swiss, other), country of birth (Switzerland, other), and civil status. Since participants and non-participants differed according to sex, age, nationality, country of birth, and civil status, participants were weighted to obtain a representative sample of the Swiss general population. Participants were weighted according to the distribution of sex, age, and nationality in all eligible persons (*n* = 5644). We used multivariable logistic regression with being a participant as outcome variable (1 = participant, 0 = eligible sample) and sex, age (six categories; 18–25, 26–35, 36–45, 46–55, 56–65, ≥ 66 years), and nationality (SFSO data; Swiss, foreigner) as explanatory variables and a multiplicative transformation to calculate appropriate weights. The multiplicative transformation consisted of multiplying the weights obtained from the regression analysis by the number of participants (*n* = 1209) and dividing them by the number of persons in the eligible sample (*n* = 5644). The weights for the participants were therefore calculated as follows: weight = (1/*predicted value*)*(1209/5644), where *predicted value* is the probability of a positive outcome in the logistic regression. All analyses were conducted taking into consideration those weights and applying the *survey* command in Stata. This command fits statistical models for survey data by adjusting the results of a command for previously defined survey settings, i.e. the weights for the participants [30].

The SF-36 has been developed on the basis of principal component analysis [24] and, consequently, the majority of studies conducted on the SF-36 based their analyses on the assumption of a reflective model, i.e. items being effects of the theoretical constructs (subscales).

### Validation of the SF-36v2 questionnaire in Switzerland

To investigate if the SF-36v2 questionnaire is valid in Switzerland, we assessed scaling assumptions, reliability, and validity of the SF-36v2.

#### Scaling assumptions

We tested if the variances of the items and the item-subscale correlations corrected for item-subscale overlap (i.e. item-rest correlations) were similar within each of the eight health domain subscales.

#### Reliability

To assess internal consistency, we calculated Cronbach’s alpha and item-subscale correlations. Cronbach’s alpha of > 0.70 [22, 23] and item-rest correlations > 0.40 were considered satisfactory [24]. Reliability of the summary measures PCS and MCS was calculated taking into account the reliability of each of the eight health domain subscales, the covariances among them, and the factor score coefficients [2].

#### Validity

Construct validity was assessed using principal component analysis, item-subscale correlations (item-rest correlations for the subscales and their respective items), and interscale correlations (Pearson and Spearman correlations) between the health domain subscales and the two summary measures PCS and MCS. If the correlation between an item and its respective subscale (item-rest correlation) is significantly higher than its correlation with the other subscales (item-subscale correlations), its inclusion in that hypothetical subscale is supported. If the correlation between two subscales is less than their reliability coefficients (Cronbach’s alpha), there is evidence of unique reliable variance measured by the respective subscale.

### SF-36v2 health domain subscales

We calculated descriptive statistics for the eight health domain subscales (*p* scores) for the whole study sample and the different subsamples according to sex, age, migration background, education, employment, partnership, civil status, children in household, chronic condition or health problem, and questionnaire language. We tested the differences in means of health domain subscales for the different subsamples using Wald tests (global test). Except for age, Wald tests were performed without and with adjustment for age since we assumed correlations between age and other covariates.

### SF-36v2 summary measures PCS and MCS

To obtain the weights to calculate the summary measures PCS and MCS, we calculated factor score coefficients applying principal component analysis followed by orthogonal varimax rotation as proposed in the SF-36v2 manual [24]. We also calculated the proportion of variance in the health domain subscales explained (i.e. communality) and not explained (i.e. uniqueness) by the factors.

Using the factor score coefficients, the summary measures PCS and MCS were calculated as weighted sums of the health domain subscales (*p* scores). The obtained *p* scores were converted into *T* scores with a mean of 50 and a standard deviation of 10. We analysed *T* scores of the summary measures for the whole study sample and for the different subsamples. The differences in means of summary measures for the different subsamples were tested applying Wald tests. Wald tests were performed without and with adjustment for age. Additionally, we conducted multivariable regression analyses for PCS and MCS, respectively. We included characteristics that were significantly (*p* < 0.05) associated with PCS and MCS, respectively, when adjusting for age.

We compared our factor score coefficients with those from other countries (United States (USA) [27], Germany [31], United Kingdom (UK) [32], New Zealand [33], and Australia [34]). We calculated summary measures in two different ways: (i) with Swiss health domain subscale *p* scores and country-specific factor score coefficients (referred to as *PCS* and *MCS Switzerland*), and (ii) with country-specific health domain subscale p scores and country-specific factor score coefficients (referred to as *PCS* and *MCS Other*). For Switzerland, we used person-level *p* scores to calculate PCS and MCS Switzerland. For the other countries, we used mean *p* scores to calculate PCS and MCS Other.

Statistical analyses were carried out using Stata version 14.2 (StataCorp LP, College Station, Texas, USA) and R (The R Project for Statistical Computing, R for Windows 3.3.2).

## Results

### Study sample

**Fig. 1:**
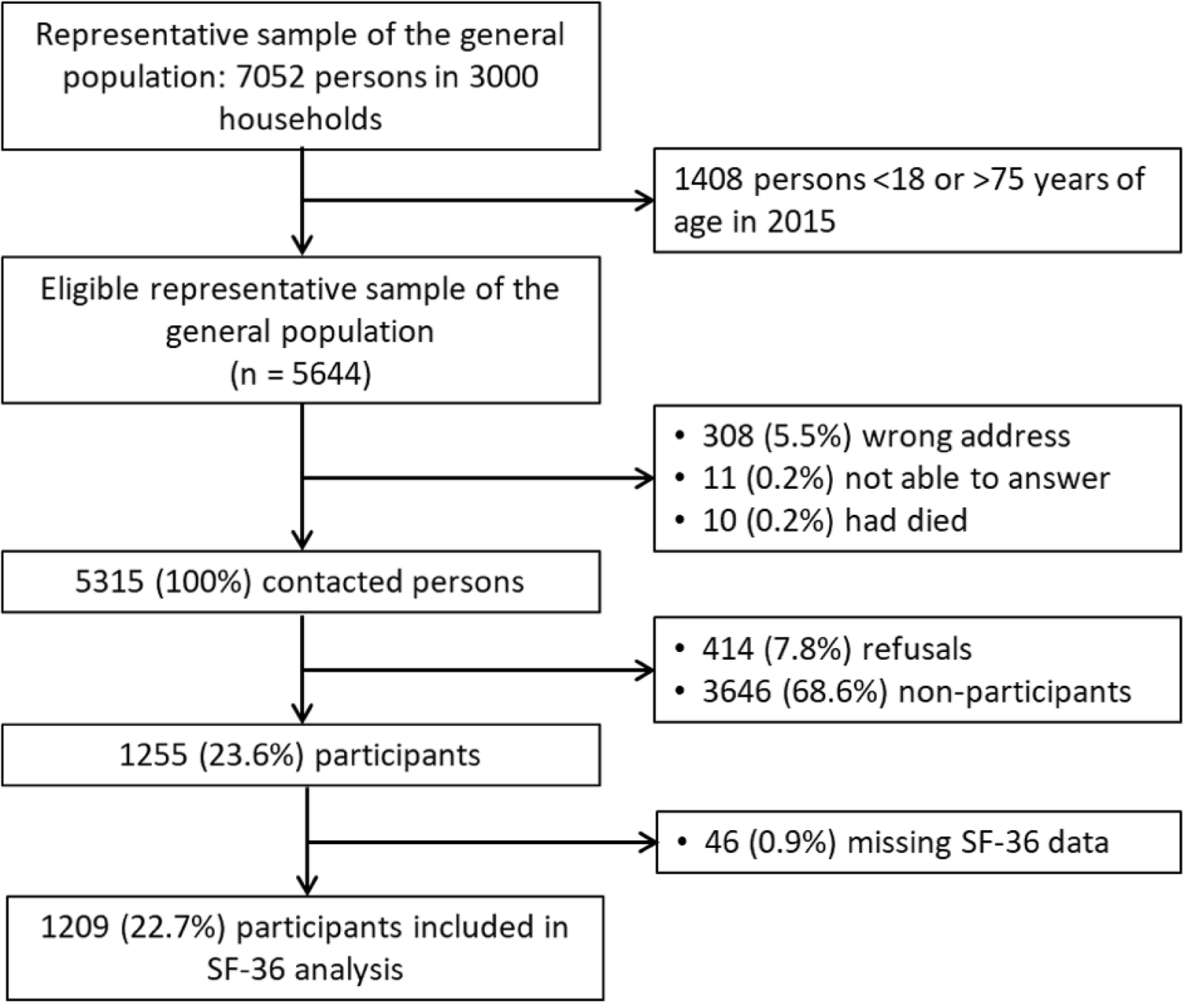
Flow chart of the study sample

Of 7052 persons from 3000 households obtained from the SFSO, 5644 were aged between 18 and 75 years in 2015 and eligible for the study (Table 1; Fig. 1). Of those, 308 persons (5.5%) were not living at the indicated address, 11 (0.2%) were not able to answer, i.e. not speaking German, French, or Italian, or too ill to participate, and 10 (0.2%) had died resulting in a sample of 5315 contacted persons. Of those, 1209 (22.7%) completed the SF-36v2 questionnaire. They were on average aged 48.7 years and 58.1% were women (Table 1).

**Table 1:** Comparison of SF-36v2 participants and non-participants of the contacted sample of the general population and characteristics of the eligible sample of the general population

	Contacted sample of the general population (*n* = 5315)			Eligible^e^ sample of the general population (*n* = 5644)
	SF-36v2 participants (*n* = 1209)	Non-participants (*n* = 4106)	*p* Value^b^	
	*n* (%)	*n* (%)		*n* (%)
Sex			< **0.001**	
Male	507 (41.9)	2145 (52.2)		2822 (50.0)
Female	702 (58.1)	1961 (47.8)		2822 (50.0)
Age at study (years)			< **0.001**	
18–25	92 (7.6)	416 (10.1)		551 (9.8)
26–35	164 (13.6)	767 (18.7)		1053 (18.7)
36–45	231 (19.1)	746 (18.2)		1039 (18.4)
46–55	278 (23.0)	909 (22.1)		1234 (21.8)
56–65	232 (19.2)	708 (17.3)		967 (17.1)
66–75	212 (17.5)	560 (13.6)		800 (14.2)
Nationality (SFSO)^a^			< **0.001**	
Swiss	1053 (87.1)	3014 (73.4)		4257 (75.4)
Foreigner	156 (12.9)	1091 (26.6)		1385 (24.5)
Country of birth (SFSO)^a^			< **0.001**	
Switzerland	976 (80.7)	2666 (64.9)		3809 (67.5)
Other country	229 (18.9)	1413 (34.4)		1803 (32.0)
Migration background				
No	948 (78.4)	–		–
Yes	261 (21.6)	–		–
Educational achievement^a^			n.a.^c^	
Compulsory schooling	95 (7.9)	–		–
Vocational training	554 (45.8)	–		–
Upper secondary education	206 (17.0)	–		–
University education	288 (23.8)	–		–
Employment status^a^			n.a.^c^	
Employed	822 (68.0)	–		–
Unemployed	144 (11.9)	–		–
Retired	212 (17.5)	–		–
Partnership^a^			n.a.^c^	
Yes	913 (75.5)	–		–
No	258 (21.3)	–		–
Civil status (SFSO)			**0.010**	
Single	393 (32.5)	1452 (35.4)		2010 (35.6)
Married	646 (53.4)	2198 (53.5)		2972 (52.7)
Divorced or widowed	170 (14.1)	456 (11.1)		662 (11.7)
Civil status (questionnaire)			n.a.^c^	
Single	354 (29.3)	–		–
Married	617 (51.0)	–		–
Divorced or widowed	182 (15.1)	–		–
Children (≤ 14 years of age) in household^a^			n.a.^c^	
No	885 (73.2)	–		–
Yes	258 (21.3)	–		–
Number of children (≤ 14 years of age) in household^a^			n.a.^c^	
0	885 (73.2)	–		–
1	106 (8.8)	–		–
2	120 (9.9)	–		–
> 2	32 (2.7)	–		–
Chronic condition or health problem^a^			n.a.^c^	
No	709 (58.6)	–		–
Yes	489 (40.5)	–		–
Questionnaire language^d^			0.104	
German	888 (73.4)	3001 (73.1)		4075 (72.2)
French	261 (21.6)	904 (22.0)		1283 (22.7)
Italian	60 (5.0)	201 (4.9)		286 (5.1)

	Mean (SD)	Mean (SD)	*p* value^b^	
Age at study	48.7 (15.2)	46.0 (15.5)	< **0.001**	46.2 (15.5)

*p* Values < 0.05 are indicated in bold

*SF-36v2* short form-36 version 2, *n.a*. not available/applicable, *n* number, *SD* standard deviation, *SFSO* Swiss Federal Statistical Office

^a^Missing values; percentages are based on the total number of participants/non-participants

^b^*p* Value calculated from Chi-square test statistics (categorical variables) or *t* test statistics (continuous variables) comparing participants and non-participants

^c^Information was not available for non-participants

^d^For non-participants and the eligible sample of the general population, questionnaire language refers to the language of the information letter sent

^e^Eligibility criteria: Swiss resident and aged between 18 and 75 years in 2015

### Validation of the SF-36v2 questionnaire in Switzerland

Assessing scaling assumptions, we found similar variances among the items of the health domain subscales and similar item-rest correlations.

Regarding reliability, Cronbach’s alpha coefficients were satisfactory for all health domain subscales (Table 2). Item-rest correlations were satisfactory apart from items 2 and 4 of the general health perceptions subscale (0.38 and 0.39). Reliability of the summary measures was 0.94 for PCS and 0.93 for MCS.

Regarding validity, the principal component analysis revealed two factors with eigenvalue > 1 indicating a two-factor structure. Differences between item-rest correlations and item-subscale correlations were satisfactory except for six items. Correlations between the subscales were lower than their respective Cronbach’s alpha indicating unique reliable variance.

### SF-36v2 health domain subscales

The number of imputed values per item due to missing values ranged from 0 to 11 (0.9% of the 1209 completed SF-36v2 questionnaires). Results were similar for imputed and non-imputed health domain subscale scores. We therefore used the imputed subscales for the analyses.

Descriptive statistics of the health domain subscale scores for the whole sample are displayed in Table 2. The ceiling effect was high for the subscales Physical Functioning, Physical Role Functioning, Bodily Pain, Social Role Functioning, and Emotional Role Functioning (38–59%, Table 2). For the different subsamples, they are displayed in Table 3 and Tables S1–S9 in Online Resource. Health domains related to physical health (PF, RP, BP, GH) were better in younger persons, whereas health domains related to mental health (VT, SF, RE, MH) were better in older persons (Table S2). All health domains but PF were better in men than in women (Table S1). Regarding language regions, VT was better in Italian speaking persons, whereas the other health domains were better in German speaking persons (Table 3).

**Table 2:** SF-36v2 health domain subscales: mean p scores with 95% confidence interval, standard deviation, percentage floor, percentage ceiling, rotated factor loadings, uniqueness, communality, Cronbach’s alpha (*n* = 1209)

Scale	Mean *p* score	95% CI	SD	Percentage floor [%]	Percentage ceiling [%]	Rotated factor loading		Uniqueness	Communality	Cronbach’s alpha
						PCS	MCS			
Physical Functioning (PF)	91.16	(90.19, 92.13)	17.01	0.58	48.72	0.81	0.13	0.33	0.67	0.92
Physical Role Functioning (RP)	86.41	(85.22, 87.60)	20.60	0.74	49.71	0.76	0.41	0.25	0.75	0.92
Bodily Pain (BP)	74.58	(73.05, 76.10)	26.03	0.00	38.96	0.78	0.16	0.37	0.63	0.92
General Health Perceptions (GH)	75.64	(74.62, 76.65)	17.35	0.17	4.47	0.67	0.43	0.36	0.64	0.73
Vitality (VT)	63.24	(62.22, 64.26)	17.22	0.25	1.90	0.33	0.78	0.29	0.71	0.81
Social Role Functioning (SF)	85.84	(84.66, 87.03)	20.02	0.41	54.43	0.30	0.81	0.26	0.74	0.86
Emotional Role Functioning (RE)	87.64	(86.50, 88.78)	19.22	0.74	58.97	0.21	0.81	0.31	0.69	0.90
Mental Health (MH)	75.02	(74.07, 75.98)	16.18	0.25	3.97	0.14	0.90	0.18	0.82	0.84

*CI* confidence interval, *SD* standard deviation, *PCS* physical component summary, *MCS* mental component summary

**Table 3:** SF-36 v2 health domain subscales: mean (*p* scores) with 95% confidence interval, standard deviation, *p* values from Wald test (global test) without and with adjustment for age according to questionnaire language (German, French, Italian)

Scale	German			French			Italian				
	Mean *p* score	95% CI	SD	Mean *p* score	95% CI	SD	Mean *p* score	95% CI	SD	*p* Value	
										Crude	Adjusted for age
Physical Functioning (PF)	92.24	(91.28, 93.19)	14.75	87.87	(85.05, 90.69)	22.06	90.57	(86.11, 95.03)	17.05	**0.014**	**0.007**
Physical Role Functioning (RP)	87.60	(86.32, 88.88)	19.62	82.88	(79.89, 85.88)	23.07	85.28	(79.99, 90.58)	19.75	**0.015**	**0.011**
Bodily Pain (BP)	77.70	(75.99, 79.41)	25.65	65.53	(62.27, 68.79)	25.02	70.81	(63.18, 78.44)	25.76	< **0.001**	< **0.001**
General Health Perceptions (GH)	76.57	(75.38, 77.75)	17.45	73.05	(70.82, 75.29)	17.29	74.01	(70.00, 78.01)	14.75	**0.017**	**0.012**
Vitality (VT)	64.79	(63.67, 65.92)	16.87	57.43	(55.08, 59.77)	17.66	66.88	(62.80, 70.96)	14.11	< **0.001**	< **0.001**
Social Role Functioning (SF)	88.46	(87.18, 89.75)	19.15	78.27	(75.45, 81.09)	20.90	82.55	(77.74, 87.35)	18.52	< **0.001**	< **0.001**
Emotional Role Functioning (RE)	89.52	(88.32, 90.73)	17.93	82.81	(79.93, 85.70)	21.81	82.59	(77.42, 87.77)	18.69	< **0.001**	< **0.001**
Mental Health (MH)	77.47	(76.49, 78.46)	14.62	67.18	(64.77, 69.59)	18.36	75.25	(71.44, 79.06)	14.87	< **0.001**	< **0.001**

*p* Values are obtained from Wald test without (crude) and with adjustment for age (adjusted for age). *p* values < 0.05 are indicated in bold

*CI* confidence interval, *SD* standard deviation

### SF-36v2 summary measures PCS and MCS

The health domains PF, RP, BP, and GH showed high loadings for the physical health component, whereas VT, SF, RE, and MH showed high loadings for the mental health component, indicating greatest physical and mental health content, respectively (Table 2). The communalities of the health domain subscales ranged from 0.63 to 0.82 (Table 2). The proportion of explained reliable variance was 81.9%.

**Fig. 2:**
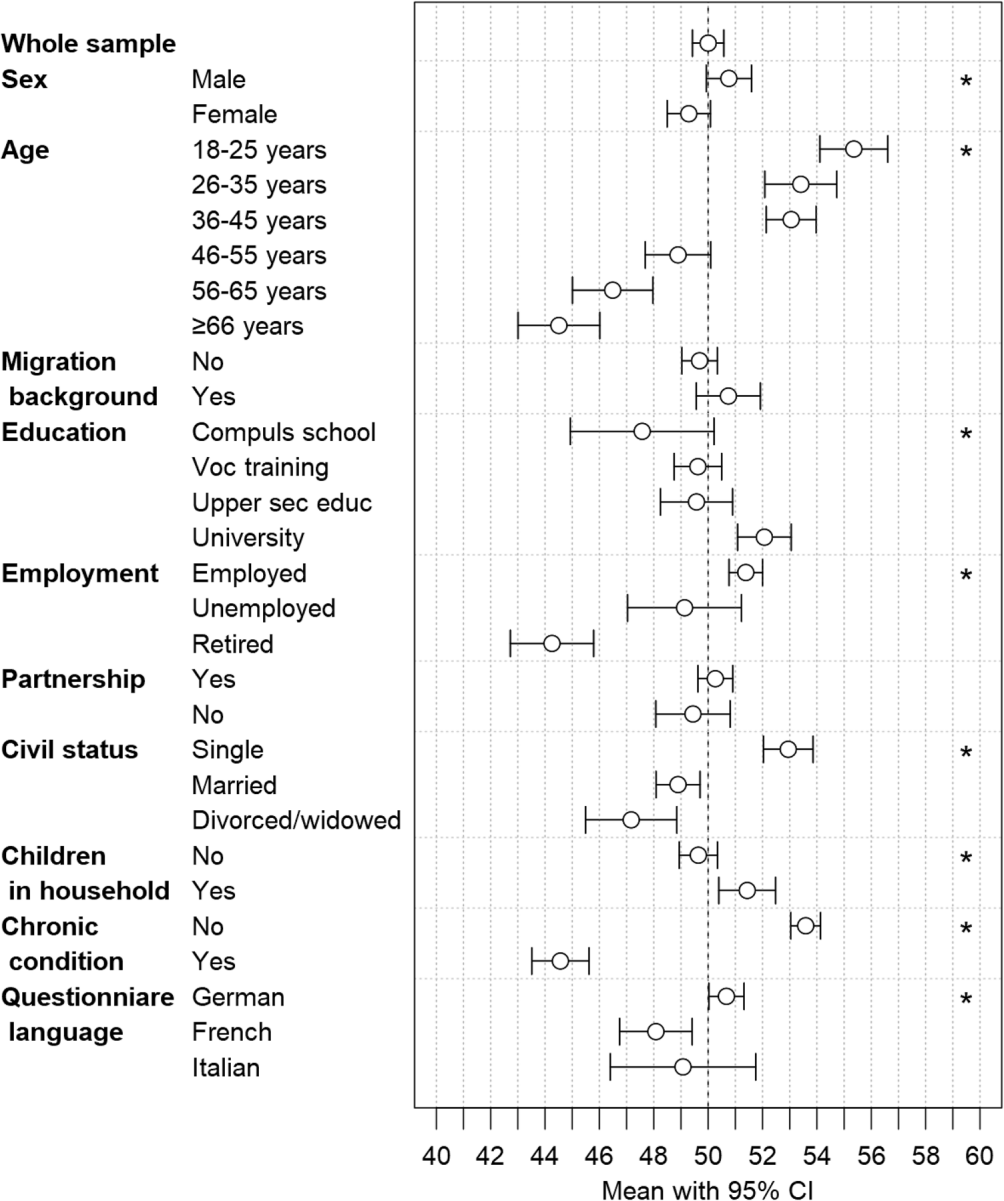
PCS: mean with 95% confidence interval for the whole study sample and subsamples according to sex, age, migration background, educational achievement, employment status, partnership, civil status, children in household, presence of chronic condition or health problem, and questionnaire language. The vertical dashed line indicates a mean of 50.00, and stars indicate *p* values < 0.05 from Wald test (global test). *PCS* physical component summary, *CI* confidence interval, *Compuls school* compulsory schooling, *Voc training* vocational training, *Upper sec educ* upper secondary education, *University* university education

We found better physical health (PCS) in men (crude *p* = 0.012) and younger persons (*p* < 0.001) (Fig. 2, Table S10). Physical health was also better in persons with higher attained education (*p* < 0.001), employed persons (*p* < 0.001), single persons (*p* < 0.001), persons with children in the household, and in German speaking persons (*p* = 0.002). Significant differences for civil status and children in the household diminished with adjustment for age (Table S10).

**Fig. 3:**
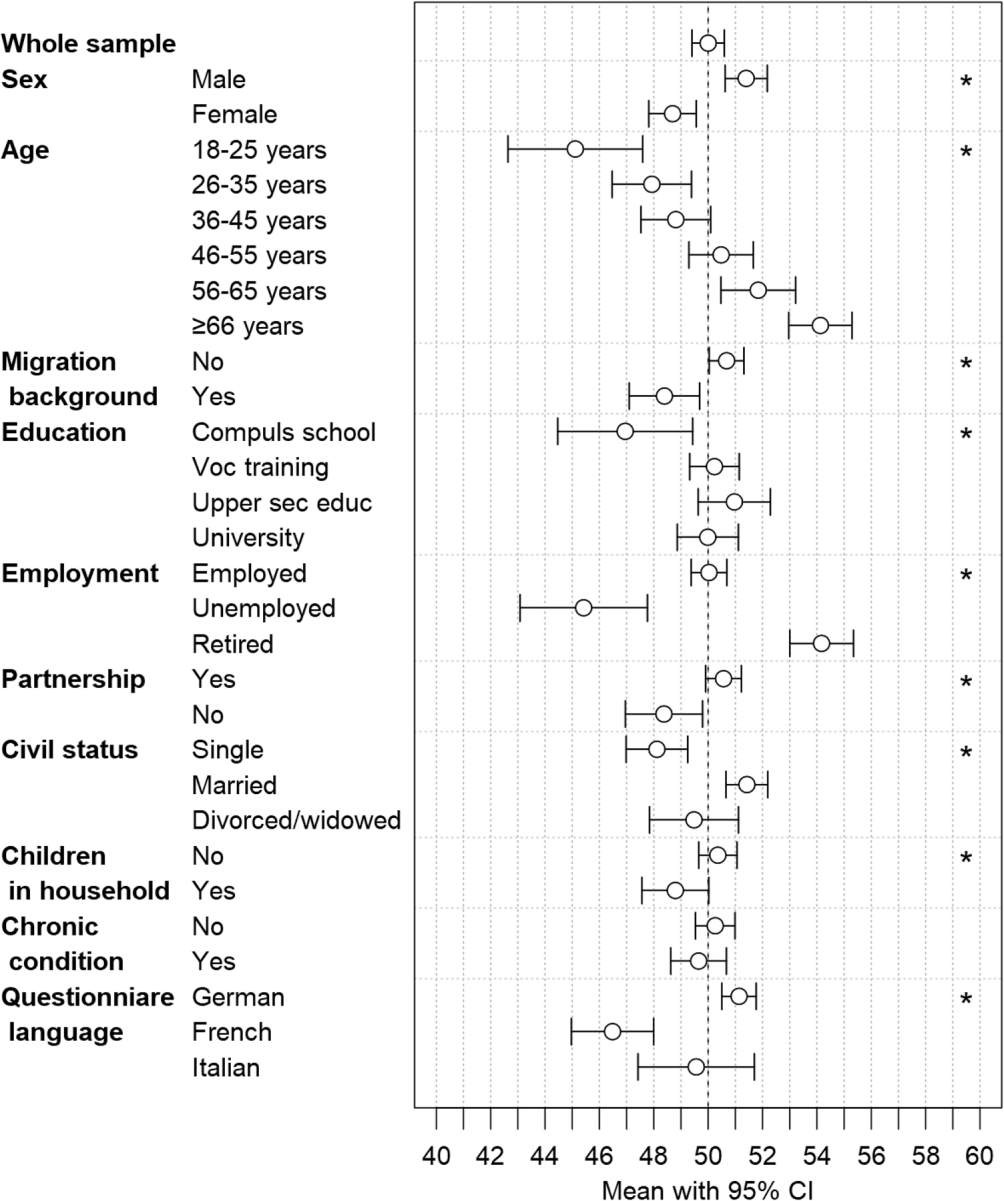
MCS: mean with 95% confidence interval for the whole study sample and subsamples according to sex, age, migration background, educational achievement, employment status, partnership, civil status, children in household, presence of chronic condition or health problem, and questionnaire language. The vertical dashed line indicates a mean of 50.00, and stars indicate *p* values < 0.05 from Wald test (global test). *MCS* mental component summary, CI confidence interval, *Compuls school* compulsory schooling, *Voc training* vocational training, *Upper sec educ* upper secondary education *University* university education

Mental health (MCS) was better in men (*p* < 0.001) and in older persons (*p* < 0.001) (Fig. 3, Table S10). Furthermore, we found better mental health in persons without migration background (*p* = 0.002), retired persons (*p* < 0.001), persons living in a partnership (*p* = 0.006), persons without children in the household (*p* = 0.030), and in German speaking persons (*p* < 0.001). Significant differences in mental health diminished with adjustment for age for education, partnership, and children in the household (Table S10).

Considering significantly associated characteristics together in multivariable analyses, we found better physical health in younger persons (*p* < 0.001), persons with higher attained education (*p* = 0.036), persons without chronic health conditions (*p* < 0.001), and German speaking persons (*p* = 0.003) (Table S11). Mental health was found to be better in men (*p* = 0.003), older persons (*p* < 0.001), employed and retired persons (*p* = 0.035), persons without chronic health conditions (*p* = 0.018), and German speaking persons (*p* < 0.001) (Table S11).

Compared to other countries, we found better physical health (Table 4) and worse mental health (Table 5) in Switzerland.

**Table 4:** SF-36v2 health domain subscales: factor score coefficients and means (*p* scores) for Switzerland, USA, Germany, UK, New Zealand, and Australia; and PCS calculated with the Swiss sample data (person-level *p* scores; PCS Switzerland) or the respective country data (mean *p* scores; PCS Other) and the respective country-specific factor score coefficients

	Switzerland		USA		Germany		UK		New Zealand		Australia	
	Factor score coefficient	Mean* p* score	Factor score coefficient [15]	Mean *p* score [24]	Factor score coefficient [31]	Mean *p* score [10]	Factor score coefficient [32]	Mean *p* score [32]	Factor score coefficient [33]	Mean *p* score [33]	Factor score coefficient [34]	Mean *p* score [34]
Time of data collection	2015–2016	2015–2016	1990	2009	1997–1999	2008–2011	1997	1997	2006–2007	2006–2007	2004	2004
Physical Functioning (PF)	0.434	91.16	0.424	79.15	0.451	86.60	0.456	87.99	0.397	85.90	0.409	84.64
Physical Role Functioning (RP)	0.324	86.41	0.351	79.13	0.310	82.10	0.362	87.17	0.367	85.70	0.325	84.41
Bodily Pain (BP)	0.406	74.58	0.318	65.60	0.375	74.80	0.367	78.80	0.340	75.30	0.289	76.45
General Health Perceptions (GH)	0.262	75.64	0.250	64.65	0.286	69.30	0.199	71.06	0.150	74.50	0.231	71.90
Vitality (VT)	− 0.046	63.24	0.029	57.69	0.028	61.60	− 0.050	58.04	0.028	64.00	0.106	61.12
Social Role Functioning (SF)	− 0.070	85.84	− 0.008	82.75	− 0.047	86.10	− 0.028	82.77	0.050	88.40	0.014	86.19
Emotional Role Functioning (RE)	− 0.125	87.64	− 0.192	86.42	− 0.156	86.00	− 0.110	85.75	− 0.131	93.70	− 0.183	91.59
Mental Health (MH)	− 0.194	75.02	− 0.221	75.00	− 0.105	72.90	− 0.256	71.92	− 0.225	82.30	− 0.205	80.63

Mean (SD)												
PCS Switzerland	83.25 (20.80)		79.35 (19.60)		93.69 (21.25)		80.86 (20.29)		82.31 (19.51)		80.88 (18.94)	
PCS other	83.25 (20.80)		66.20		88.99		81.68		77.75		75.15	

For a detailed description of the samples see Table S12 in Online Resource

*SD* standard deviation, *PCS* physical component summary, *MCS* mental component summary

**Table 5:** SF-36v2 health domain subscales: factor score coefficients and means (*p* scores) for Switzerland, USA, Germany, UK, New Zealand, and Australia; and MCS calculated with the Swiss sample data (person-level p scores; MCS Switzerland) or the respective country data (mean *p* scores; MCS Other) and the respective country-specific factor score coefficients

	Switzerland		USA		Germany		UK		New Zealand		Australia	
	Factor score coefficient	Mean *p* score	Factor score coefficient [15]	Mean *p* score [24]	Factor score coefficient [31]	Mean *p* score [10]	Factor score coefficient [32]	Mean *p* score [32]	Factor score coefficient [33]	Mean *p* score [33]	Factor score coefficient [34]	Mean *p* score [34]
Time of data collection	2015–2016	2015–2016	1990	2009	1997–1999	2008–2011	1997	1997	2006–2007	2006–2007	2004	2004
Physical Functioning (PF)	− 0.184	91.16	− 0.230	79.15	− 0.192	86.60	− 0.227	87.99	− 0.160	85.90	− 0.224	84.64
Physical Role Functioning (RP)	− 0.037	86.41	− 0.123	79.13	− 0.041	82.10	0.102	87.17	− 0.097	85.70	− 0.096	84.41
Bodily Pain (BP)	− 0.158	74.58	− 0.097	65.60	− 0.092	74.80	− 0.130	78.80	− 0.123	75.30	− 0.105	76.45
General Health Perceptions (GH)	0.003	75.64	− 0.016	64.65	− 0.006	69.30	0.036	71.06	0.110	74.50	0.001	71.90
Vitality (VT)	0.274	63.24	0.235	57.69	0.284	61.60	0.278	58.04	0.257	64.00	0.157	61.12
Social Role Functioning (SF)	0.296	85.84	0.269	82.75	0.338	86.10	0.272	82.77	0.212	88.40	0.249	86.19
Emotional Role Functioning (RE)	0.325	87.64	0.434	86.42	0.360	86.00	0.329	85.75	0.390	93.70	0.449	91.59
Mental Health (MH)	0.390	75.02	0.486	75.00	0.390	72.90	0.460	71.92	0.491	82.30	0.476	80.63

Mean (SD)												
MCS Switzerland	68.91 (17.91)		72.41 (19.41)		79.42 (19.40)		85.42 (20.18)		81.64 (19.29)		69.89 (18.52)	
MCS Other	68.91 (17.91)		74.44		78.70		91.42		89.02		75.54	

For a detailed description of the samples see Table S12 in Online Resource

*SD* standard deviation, *PCS* physical component summary, *MCS* mental component summary

## Discussion

We found the SF-36v2 questionnaire to be a valid and reliable instrument to evaluate HRQOL in Switzerland. Men reported better HRQOL than women. Physical health was better in younger persons and mental health was better in older persons. Furthermore, physical health was better in persons with higher education, whereas mental health was better in employed and retired persons. Regarding language regions, physical and mental health were better in German speaking persons compared to French or Italian speaking persons. Compared to other countries, we found better physical health and worse mental health in Switzerland.

### Validation of the SF-36v2 questionnaire in Switzerland

The SF-36v2 questionnaire showed good reliability and validity in Switzerland. The high ceiling effect for five (PF, RP, BP, SF, and RE) of eight health domain subscales indicate very good HRQOL in these health domains for the majority of persons included in our sample.

### Physical and mental health

In line with our findings, other studies from New Zealand and Australia [9], Germany [10], Brazil [6, 7], and Norway [13] found better HRQOL in men than in women. In a Spanish wage-earning population, men had better mean mental health, but also a higher prevalence of poor mental health than women [4]. Physical health was worse for older persons in our study and studies from New Zealand and Australia [9], Germany [10], Brazil [6, 7], Sweden [5], and Norway [13]. Results on mental health and age differ between countries: our and other studies from New Zealand and Australia [9], Germany [10], and Norway [13] found better mental health in older persons, but in Spain [4], mental health decreased with age, and in Sweden [5] and Brazil [6, 7], mental health first increased with age and decreased again for the elderly.

We found better physical health for persons with higher education similar to studies in Sweden [5], Brazil [6], Spain [4], and Norway [13]. In Finland, persons with higher education reported better physical health, but worse mental health [8]. In Spain, workers with lower educational attainment had a higher prevalence of poor mental health [4]. In our study, employed and retired persons reported better mental health compared to unemployed persons. In Spanish workers, prevalence of poor mental health was higher among manual workers than non-manual workers and among those who had been unemployed previously, and prevalence of poor mental health increased with increasing employment precariousness [4]. In Finland, persons in a higher occupational class reported better physical and mental health than persons in a lower occupational class [8]. Retired persons in our study reported better mental health than employed and unemployed persons. A review on longitudinal studies reported consistently better mental health in retired persons and inconsistent findings for physical health after retirement [35]. Reasons for better mental health in retired persons might be reduced to work-related duties and stress [35]. Reasons for better physical health might be a healthier life style after retirement [35]; conversely, there are also reasons for worse physical health: reduction of physical and mental demands due to loss of work and a less healthy life style [35]. The conflicting results for physical health might also be due to methodological problems such as confounding or reverse causality [35, 36]. A study conducted in England approaching these problems found that retirement increased the risk for the diagnosis of several health conditions and poor self-rated health [36]. A study in more than 23,000 persons aged ≥ 50 years from 19 European countries found that the partner’s retirement decreased moderate physical activity, increased the frequency and the amount of alcohol consumption, and had a negative impact on self-rated health [37]. Own retirement increased physical activity, had no impact on smoking, increased the frequency of alcohol consumption, and had a positive effect on health [37].

Education and employment are proxies for socioeconomic position of persons in the society. In this light, our findings are in line with studies in Sweden [5], New Zealand and Australia [9], Germany [10], and France [11] showing better physical and mental health for persons with higher socioeconomic position. In the Netherlands, persons aged ≥ 55 years with higher socioeconomic position had better physical health and a lower risk of a decline of mental health over 7 years, but socioeconomic position was not associated with mental health or a decline in physical health over 7 years [12]. In Finland, physical health was better in persons with higher socioeconomic status and better material circumstances, whereas mental health was found to be better in persons with lower socioeconomic status and better material circumstances [8]. This study population only included employed persons and the authors hypothesize that the association between lower socioeconomic status and better mental health might be due to higher work demands or mental strains among persons with higher socioeconomic status and under-reporting of minor mental health problems among persons with lower socioeconomic position.

Chronically ill persons reported worse physical and mental health than persons without health problems. Also many other studies reported that chronic conditions [6], health events [38], and a diversity of diseases [27] impaired physical health.

Persons living in the German speaking part of Switzerland reported better physical and mental health than persons living in the French and Italian speaking part. This could be explained by different patterns of health behaviours in Switzerland: persons ≥ 30 years in the German speaking part of Switzerland were physically more active and smoked less [39], and they were less often unemployed and had less often only basic education compared to persons in the French and Italian speaking part [40]. Compared to the sample of young adults living in the French speaking part of Switzerland [17], our French speaking subsample reported lower Physical Functioning, Physical Role Functioning, Bodily Pain, General Health Perceptions, Vitality, and Social Role Functioning, better Emotional Role Functioning, and similar Mental Health. These differences are likely to be explained by more women (59% vs. 53%) and the older age (mean age = 49 years vs. 30 years) in our subsample.

Age had a major influence on both physical and mental health. Adjustment for age reduced differences in physical health for civil status and children in the household. Thus, better physical health in single persons and persons with children in the household might partly be explained by the fact that single persons and persons with children in the household were younger in our sample. Differences in mental health were reduced for education, partnership, and children in the household suggesting that these differences may be partly explained by age.

Swiss persons reported better physical health and worse mental health than persons from other countries. Countries differ in socioeconomic characteristics [18] known to be related to HRQOL and, thus, these differences might be reflected by differences in HRQOL. Differences in stigmatization of mental illnesses between countries might contribute to differences in reported mental health [41]. Switzerland is known for a good health care system; thus, physical health might be better than in other countries. In our sample, 41% of respondents reported a chronic condition or health problem. Among respondents in the UK (37% with longstanding illness; [32]), New Zealand (66% with health condition; [33, 42, 43]), and the USA (various diseases with prevalences ranging from 1 to 38%; [24]), chronic conditions and health problems were common and might contribute to worse physical health in these samples. Our sample included 58% women, study samples in the USA (51%; [24]), Germany (53%; [10]), and Australia (51%; [34]) included less women probably explaining better mental health in these samples. However, the larger proportion of women in our sample did not result in worse physical health compared to other countries. Judged on the basis of the available information, the age distributions of the samples seem to be similar.

The differences between the *PCS* and *MCS Switzerland* estimates indicate that the choice of weighting coefficients matters and that it is therefore crucial to use country-specific weighting coefficients to investigate HRQOL measured by the SF-36. Our factor score coefficients will enable future Swiss studies on HRQOL to apply weighting coefficients derived from Swiss normative data.

### Limitations and strengths

Our questionnaire survey had a relatively low response rate of 23%, similar to other recent studies [13]. However, using weights for sociodemographic characteristics, our sample is representative for the Swiss general population. Thus, the presented normative data adequately reflect the situation in Switzerland in terms of sex, age, and nationality. Other covariates such as for example health status that were not available for non-respondents might still have affected our results. The SF-36 questionnaire is a self-report instrument being prone to reporting bias; however, the SF-36 is a widely used, reliable, and valid instrument to assess HRQOL [1, 15, 24, 27]. Social desirability bias might be present. The comparison with HRQOL in other countries is limited by the fact that time periods of data collection vary and we only included countries where p scores and corresponding weighting coefficients were available. We included a variety of covariates known to be related to HRQOL. The three language regions in Switzerland (German, French, and Italian) allowed us to investigate HRQOL in three also culturally diverse regions. We provide normative data for the Swiss general population, and also for subsamples according to a variety of sociodemographic and socioeconomic characteristics.

## Conclusions

In summary, HRQOL in Switzerland follows the same patterns as in other countries, with better HRQOL in men compared to women and worse physical and better mental health in older persons. Furthermore, physical and mental health were better in German speaking persons compared to French and Italian speaking persons. The presented normative data and weighting coefficients will enable future studies to measure HRQOL assessed by the SF-36 questionnaire using normative data and weighting coefficients based on a representative sample of the Swiss general population.

## Electronic supplementary material

Below is the link to the electronic supplementary material.

Supplementary material 1 (DOCX 123 KB)

Supplementary material 2 (DOCX 23 KB)
