# Supplementary material for: Health-related quality of life in Switzerland: normative data for the SF-36v2 questionnaire
Source: Qual Life Res. 2019 Mar 8;28(7):1963–77. doi: 10.1007/s11136-019-02161-5 (PMC6571102; doi:10.1007/s11136-019-02161-5)
Supplement: Supplementary file 1 — Supplementary material 1 (DOCX 123 KB) [file 11136_2019_2161_MOESM1_ESM.docx]

Health-related quality of life in Switzerland: normative data for the SF-36 questionnaire

Katharina Roser ^1^, Luzius Mader ^1^, Julia Baenziger ^1^, Grit Sommer ^2,3^, Claudia E. Kuehni ^2,3^ and Gisela Michel ^1,*^

^1^ Department of Health Sciences and Health Policy, University of Lucerne, Lucerne, Switzerland

^2^ Swiss Childhood Cancer Registry, Institute of Social and Preventive Medicine, University of Bern, Bern, Switzerland

^3^ Department of Paediatrics, University Children's Hospital, University of Bern, Switzerland

* Corresponding author

**Corresponding author:**

Gisela Michel, Department of Health Sciences and Health Policy, University of Lucerne, Frohburgstrasse 3, PO Box 4466, 6002 Lucerne, Switzerland, Phone: +41 41 229 59 55, Fax: +41 41 229 56 35, Email: gisela.michel@unilu.ch

**Quality of Life Research**

**Online Resource**

**Table S1:** SF-36v2 health domain subscales: mean (p scores) with 95% CI, SD, p values from Wald test (global test) without and with adjustment for age according to sex (men, women).

|  | **Men** | | | **Women** | | |  |  |
| --- | --- | --- | --- | --- | --- | --- | --- | --- |
| **Scale** | **Mean p score** | **95% CI** | **SD** | **Mean p score** | **95% CI** | **SD** | **p value** | |
|  |  |  |  |  |  |  | **crude** | **adjusted for age** |
| Physical Functioning (PF) | 91.73 | (90.22, 93.24) | 16.22 | 90.62 | (89.39, 91.86) | 17.59 | 0.265 | 0.091 |
| Physical Role Functioning (RP) | 89.20 | (87.53, 90.87) | 18.10 | 83.80 | (82.16, 85.44) | 22.60 | **<0.001** | **<0.001** |
| Bodily Pain (BP) | 77.48 | (75.29, 79.67) | 22.66 | 71.86 | (69.78, 73.94) | 28.88 | **<0.001** | **<0.001** |
| General Health Perceptions (GH) | 76.74 | (75.26, 78.22) | 15.57 | 74.60 | (73.21, 75.99) | 18.93 | **0.040** | **0.018** |
| Vitality (VT) | 66.12 | (64.73, 67.52) | 14.68 | 60.54 | (59.11, 61.97) | 19.16 | **<0.001** | **<0.001** |
| Social Role Functioning (SF) | 88.50 | (86.89, 90.12) | 16.62 | 83.35 | (81.66, 85.05) | 22.87 | **<0.001** | **<0.001** |
| Emotional Role Functioning (RE) | 89.60 | (87.99, 91.22) | 16.93 | 85.79 | (84.21, 87.38) | 21.17 | **0.001** | **0.001** |
| Mental Health (MH) | 77.46 | (76.17, 78.75) | 13.62 | 72.74 | (71.37, 74.11) | 18.24 | **<0.001** | **<0.001** |

Abbreviations: CI: confidence interval; SD: standard deviation

^a^ P values are obtained from Wald test without (crude) and with adjustment for age (adjusted for age).

P values <0.05 are indicated in bold.

**Table S2**: SF-36v2 health domain subscales: mean (p scores) with 95% CI, SD, p values from Wald test (global test) according to age (18-25, 26-35, 36-45, 46-55, 56-65, 66-75 years).

|  | **18-25 years** | | | **26-35 years** | | | **36-45 years** | | |
| --- | --- | --- | --- | --- | --- | --- | --- | --- | --- |
| **Scale** | **Mean p score** | **95% CI** | **SD** | **Mean p score** | **95% CI** | **SD** | **Mean p score** | **95% CI** | **SD** |
|  |  |  |  |  |  |  |  |  |  |
| Physical Functioning (PF) | 98.18 | (97.42, 98.94) | 3.44 | 95.40 | (93.36, 97.43) | 12.36 | 95.37 | (94.04, 96.70) | 10.78 |
| Physical Role Functioning (RP) | 90.92 | (87.35, 94.49) | 15.24 | 89.71 | (87.00, 92.41) | 15.27 | 91.11 | (89.14, 93.09) | 15.10 |
| Bodily Pain (BP) | 83.42 | (78.99, 87.86) | 19.55 | 81.42 | (77.64, 85.20) | 20.70 | 80.53 | (77.54, 83.52) | 22.82 |
| General Health Perceptions (GH) | 79.83 | (76.68, 82.98) | 14.18 | 78.77 | (75.93, 81.60) | 15.69 | 77.68 | (75.51, 79.85) | 15.79 |
| Vitality (VT) | 57.31 | (53.71, 60.90) | 15.39 | 61.64 | (58.88, 64.40) | 15.26 | 62.13 | (59.98, 64.29) | 15.95 |
| Social Role Functioning (SF) | 81.90 | (77.02, 86.78) | 21.22 | 84.82 | (81.83, 87.82) | 16.73 | 85.76 | (83.14, 88.39) | 18.78 |
| Emotional Role Functioning (RE) | 83.77 | (79.24, 88.30) | 19.12 | 86.23 | (83.35, 89.10) | 15.59 | 88.63 | (86.27, 90.99) | 17.14 |
| Mental Health (MH) | 70.28 | (66.78, 73.77) | 14.77 | 73.66 | (71.25, 76.08) | 13.32 | 74.41 | (72.32, 76.50) | 15.16 |

|  | **46-55 years** | | | **56-65 years** | | | **≥66 years** | | |  |
| --- | --- | --- | --- | --- | --- | --- | --- | --- | --- | --- |
| **Scale** | **Mean p score** | **95% CI** | **SD** | **Mean p score** | **95% CI** | **SD** | **Mean p score** | **95% CI** | **SD** | **p value** |
|  |  |  |  |  |  |  |  |  |  |  |
| Physical Functioning (PF) | 91.16 | (89.17, 93.15) | 16.55 | 84.84 | (81.73, 87.95) | 23.32 | 83.81 | (81.10, 86.52) | 22.21 | **<0.001** |
| Physical Role Functioning (RP) | 86.66 | (84.12, 89.20) | 21.50 | 82.25 | (79.07, 85.42) | 25.24 | 78.18 | (74.91, 81.46) | 25.79 | **<0.001** |
| Bodily Pain (BP) | 69.86 | (66.51, 73.22) | 27.82 | 68.50 | (64.78, 72.22) | 29.05 | 67.59 | (64.07, 71.11) | 28.47 | **<0.001** |
| General Health Perceptions (GH) | 73.91 | (71.80, 76.03) | 18.03 | 73.71 | (71.32, 76.11) | 18.92 | 71.56 | (69.26, 73.85) | 18.44 | **<0.001** |
| Vitality (VT) | 63.32 | (61.24, 65.41) | 17.58 | 65.63 | (63.28, 67.98) | 18.14 | 67.30 | (64.93, 69.66) | 18.90 | **<0.001** |
| Social Role Functioning (SF) | 85.87 | (83.39, 88.35) | 20.95 | 86.91 | (84.16, 89.65) | 21.96 | 88.30 | (85.69, 90.91) | 19.72 | 0.245 |
| Emotional Role Functioning (RE) | 88.48 | (86.14, 90.83) | 19.85 | 86.86 | (83.88, 89.84) | 23.81 | 90.06 | (87.68, 92.45) | 18.29 | 0.124 |
| Mental Health (MH) | 74.55 | (72.50, 76.59) | 17.24 | 75.81 | (73.43, 78.19) | 18.72 | 80.19 | (78.26, 82.12) | 15.24 | **<0.001** |

Abbreviations: CI: confidence interval; SD: standard deviation

^a^ P values are obtained from Wald test without (crude) and with adjustment for age (adjusted for age).

P values <0.05 are indicated in bold.

**Table S3**: SF-36v2 health domain subscales: mean (p scores) with 95% CI, SD, p values from Wald test (global test) without and with adjustment for age according to migration background (no, yes).

|  | **No migration background** | | | **Migration background** | | |  |  |
| --- | --- | --- | --- | --- | --- | --- | --- | --- |
| **Scale** | **Mean p score** | **95% CI** | **SD** | **Mean p score** | **95% CI** | **SD** | **p value** | |
|  |  |  |  |  |  |  | **crude** | **adjusted for age** |
| Physical Functioning (PF) | 90.69 | (89.57, 91.82) | 18.62 | 92.26 | (90.38, 94.14) | 13.11 | 0.161 | 0.706 |
| Physical Role Functioning (RP) | 86.20 | (84.86, 87.55) | 22.19 | 86.90 | (84.49, 89.32) | 16.71 | 0.618 | 0.810 |
| Bodily Pain (BP) | 74.29 | (72.63, 75.95) | 27.41 | 75.25 | (71.96, 78.54) | 22.35 | 0.609 | 0.808 |
| General Health Perceptions (GH) | 76.05 | (74.92, 77.18) | 18.49 | 74.66 | (72.51, 76.80) | 14.42 | 0.261 | 0.089 |
| Vitality (VT) | 63.94 | (62.83, 65.04) | 18.15 | 61.59 | (59.39, 63.78) | 14.66 | 0.061 | 0.152 |
| Social Role Functioning (SF) | 86.64 | (85.36, 87.92) | 20.99 | 83.94 | (81.35, 86.53) | 17.25 | 0.067 | 0.110 |
| Emotional Role Functioning (RE) | 88.54 | (87.30, 89.78) | 20.29 | 85.49 | (83.03, 87.94) | 16.25 | **0.030** | **0.045** |
| Mental Health (MH) | 75.93 | (74.91, 76.95) | 16.78 | 72.88 | (70.76, 75.01) | 14.20 | **0.011** | **0.034** |

Abbreviations: CI: confidence interval; SD: standard deviation

^a^ P values are obtained from Wald test without (crude) and with adjustment for age (adjusted for age).

P values <0.05 are indicated in bold.

**Table S4**: SF-36 v2 health domain subscales: mean (p scores) with 95% CI, SD, p values from Wald test (global test) without and with adjustment for age according to educational achievement (compulsory schooling (ISCED 1-2), vocational training (ISCED 3-4), upper secondary education (ISCED 5), university education (ISCED 6-8)).

|  | **Compulsory schooling** | | | **Vocational training** | | |
| --- | --- | --- | --- | --- | --- | --- |
| **Scale** | **Mean p score** | **95% CI** | **SD** | **Mean p score** | **95% CI** | **SD** |
|  |  |  |  |  |  |  |
| Physical Functioning (PF) | 85.00 | (80.19, 89.81) | 23.84 | 90.81 | (89.38, 92.24) | 17.08 |
| Physical Role Functioning (RP) | 81.01 | (76.10, 85.92) | 23.50 | 85.88 | (84.09, 87.67) | 20.80 |
| Bodily Pain (BP) | 69.04 | (62.30, 75.79) | 31.68 | 74.20 | (71.92, 76.48) | 26.50 |
| General Health Perceptions (GH) | 71.89 | (68.07, 75.71) | 18.12 | 74.72 | (73.20, 76.23) | 17.72 |
| Vitality (VT) | 58.03 | (53.69, 62.38) | 20.83 | 63.53 | (62.04, 65.02) | 17.17 |
| Social Role Functioning (SF) | 79.64 | (74.77, 84.51) | 23.12 | 86.35 | (84.56, 88.15) | 20.70 |
| Emotional Role Functioning (RE) | 80.60 | (75.43, 85.77) | 24.23 | 87.70 | (86.01, 89.40) | 19.46 |
| Mental Health (MH) | 69.60 | (65.52, 73.68) | 19.50 | 75.08 | (73.62, 76.53) | 16.85 |

|  | **Upper secondary education** | | | **University education** | | |  |  |
| --- | --- | --- | --- | --- | --- | --- | --- | --- |
| **Scale** | **Mean p score** | **95% CI** | **SD** | **Mean p score** | **95% CI** | **SD** | **p value** | |
|  |  |  |  |  |  |  | **crude** | **adjusted for age** |
| Physical Functioning (PF) | 91.38 | (89.00, 93.76) | 17.78 | 94.71 | (93.24, 96.19) | 11.37 | **<0.001** | **<0.001** |
| Physical Role Functioning (RP) | 86.41 | (83.45, 89.36) | 22.21 | 90.27 | (88.26, 92.27) | 16.71 | **<0.001** | **0.001** |
| Bodily Pain (BP) | 73.68 | (70.25, 77.12) | 25.33 | 77.60 | (74.77, 80.44) | 23.03 | 0.066 | 0.086 |
| General Health Perceptions (GH) | 75.73 | (73.35, 78.11) | 17.54 | 78.42 | (76.48, 80.36) | 15.35 | **0.005** | **0.007** |
| Vitality (VT) | 64.25 | (61.93, 66.57) | 16.97 | 63.36 | (61.34, 65.37) | 15.79 | 0.095 | 0.181 |
| Social Role Functioning (SF) | 86.12 | (83.38, 88.87) | 20.07 | 86.80 | (84.55, 89.06) | 17.60 | 0.067 | 0.081 |
| Emotional Role Functioning (RE) | 88.89 | (86.18, 91.60) | 19.51 | 89.96 | (88.00, 91.92) | 15.13 | **0.008** | **0.007** |
| Mental Health (MH) | 77.23 | (75.17, 79.30) | 15.17 | 75.46 | (73.63, 77.29) | 14.14 | **0.012** | **0.031** |

Abbreviations: CI: confidence interval; SD: standard deviation

^a^ P values are obtained from Wald test without (crude) and with adjustment for age (adjusted for age). P values <0.05 are indicated in bold.

**Table S5**: SF-36 v2 health domain subscales: mean (p scores) with 95% CI, SD, p values from Wald test (global test) without and with adjustment for age according to employment status (employed, unemployed, retired).

|  | **Employed** | | | **Unemployed** | | | **Retired** | | |  |  |
| --- | --- | --- | --- | --- | --- | --- | --- | --- | --- | --- | --- |
| **Scale** | **Mean p score** | **95% CI** | **SD** | **Mean p score** | **95% CI** | **SD** | **Mean p score** | **95% CI** | **SD** | **p value** | |
|  |  |  |  |  |  |  |  |  |  | **crude** | **adjusted for age** |
| Physical Functioning (PF) | 93.42 | (92.39, 94.46) | 14.07 | 88.77 | (85.45, 92.10) | 20.48 | 82.83 | (79.97, 85.70) | 23.58 | **<0.001** | **<0.001** |
| Physical Role Functioning (RP) | 89.57 | (88.36, 90.77) | 16.74 | 80.08 | (75.46, 84.69) | 27.17 | 77.96 | (74.62, 81.31) | 26.47 | **<0.001** | **<0.001** |
| Bodily Pain (BP) | 76.46 | (74.68, 78.24) | 24.65 | 71.99 | (66.92, 77.07) | 28.79 | 67.46 | (63.96, 70.96) | 28.34 | **<0.001** | **0.009** |
| General Health Perceptions (GH) | 77.23 | (76.08, 78.37) | 15.75 | 72.02 | (68.33, 75.72) | 21.09 | 71.58 | (69.18, 73.99) | 19.10 | **<0.001** | **0.004** |
| Vitality (VT) | 63.37 | (62.20, 64.54) | 16.07 | 58.13 | (54.51, 61.74) | 20.47 | 66.94 | (64.58, 69.30) | 18.65 | **<0.001** | 0.095 |
| Social Role Functioning (SF) | 86.73 | (85.40, 88.06) | 18.18 | 79.12 | (74.57, 83.68) | 26.35 | 88.09 | (85.40, 90.78) | 20.52 | **0.003** | **0.014** |
| Emotional Role Functioning (RE) | 89.09 | (87.86, 90.32) | 16.62 | 78.06 | (73.45, 82.67) | 26.82 | 90.01 | (87.62, 92.40) | 18.98 | **<0.001** | **<0.001** |
| Mental Health (MH) | 75.31 | (74.22, 76.39) | 14.96 | 67.92 | (64.32, 71.52) | 20.43 | 80.31 | (78.34, 82.28) | 15.46 | **<0.001** | **<0.001** |

Abbreviations: CI: confidence interval; SD: standard deviation

^a^ P values are obtained from Wald test without (crude) and with adjustment for age (adjusted for age).

P values <0.05 are indicated in bold.

**Table S6**: SF-36 v2 health domain subscales: mean (p scores) with 95% CI, SD, p values from Wald test (global test) without and with adjustment for age according to partnership (yes, no).

|  | **Living in a partnership** | | | **Not living in a partnership** | | |  |  |
| --- | --- | --- | --- | --- | --- | --- | --- | --- |
| **Scale** | **Mean p score** | **95% CI** | **SD** | **Mean p score** | **95% CI** | **SD** | **p value** | |
|  |  |  |  |  |  |  | **crude** | **adjusted for age** |
| Physical Functioning (PF) | 91.73 | (90.65, 92.80) | 16.23 | 89.93 | (87.65, 92.22) | 18.81 | 0.164 | **0.006** |
| Physical Role Functioning (RP) | 87.45 | (86.17, 88.73) | 19.34 | 84.11 | (81.18, 87.04) | 23.15 | **0.041** | **0.003** |
| Bodily Pain (BP) | 75.17 | (73.43, 76.91) | 25.74 | 73.01 | (69.67, 76.36) | 26.41 | 0.262 | **0.032** |
| General Health Perceptions (GH) | 76.21 | (75.07, 77.35) | 16.79 | 74.10 | (71.77, 76.43) | 18.44 | 0.111 | **0.022** |
| Vitality (VT) | 64.26 | (63.12, 65.40) | 16.87 | 59.90 | (57.53, 62.27) | 18.11 | **0.001** | **0.008** |
| Social Role Functioning (SF) | 86.71 | (85.40, 88.01) | 19.34 | 83.58 | (80.70, 86.46) | 21.71 | 0.053 | 0.101 |
| Emotional Role Functioning (RE) | 88.68 | (87.42, 89.93) | 18.46 | 85.07 | (82.39, 87.75) | 20.55 | **0.017** | **0.036** |
| Mental Health (MH) | 75.99 | (74.92, 77.06) | 15.74 | 72.33 | (70.13, 74.54) | 17.24 | **0.004** | **0.019** |

Abbreviations: CI: confidence interval; SD: standard deviation

^a^ P values are obtained from Wald test without (crude) and with adjustment for age (adjusted for age).

P values <0.05 are indicated in bold.

**Table S7**: SF-36 v2 health domain subscales: mean (p scores) with 95% CI, SD, p values from Wald test (global test) without and with adjustment for age according to civil status (single, married, divorced/widowed).

|  | **Single** | | | **Married** | | | **Divorced or widowed** | | |  |  |
| --- | --- | --- | --- | --- | --- | --- | --- | --- | --- | --- | --- |
| **Scale** | **Mean p score** | **95% CI** | **SD** | **Mean p score** | **95% CI** | **SD** | **Mean p score** | **95% CI** | **SD** | **p value** | |
|  |  |  |  |  |  |  |  |  |  | **crude** | **adjusted for age** |
| Physical Functioning (PF) | 94.83 | (93.45, 96.22) | 12.59 | 90.29 | (88.92, 91.66) | 17.11 | 86.45 | (83.13, 89.76) | 23.29 | **<0.001** | 0.207 |
| Physical Role Functioning (RP) | 89.96 | (88.05, 91.87) | 16.96 | 86.50 | (84.91, 88.10) | 20.17 | 79.14 | (75.28, 82.99) | 26.62 | **<0.001** | **0.009** |
| Bodily Pain (BP) | 79.76 | (77.16, 82.36) | 23.21 | 72.11 | (69.94, 74.28) | 26.66 | 70.52 | (66.45, 74.58) | 28.52 | **<0.001** | 0.906 |
| General Health Perceptions (GH) | 78.40 | (76.58, 80.22) | 16.09 | 74.62 | (73.26, 75.98) | 16.81 | 72.40 | (69.59, 75.21) | 19.86 | **<0.001** | 0.363 |
| Vitality (VT) | 61.25 | (59.32, 63.17) | 16.39 | 64.72 | (63.37, 66.06) | 16.88 | 62.21 | (59.34, 65.09) | 19.64 | **0.010** | 0.141 |
| Social Role Functioning (SF) | 84.45 | (82.18, 86.72) | 19.38 | 87.23 | (85.66, 88.81) | 19.59 | 84.51 | (81.28, 87.73) | 22.49 | 0.085 | 0.227 |
| Emotional Role Functioning (RE) | 86.83 | (84.76, 88.91) | 17.75 | 89.49 | (87.99, 90.99) | 18.62 | 83.90 | (80.54, 87.25) | 22.88 | **0.005** | **0.006** |
| Mental Health (MH) | 73.65 | (71.91, 75.39) | 14.90 | 76.53 | (75.27, 77.79) | 15.63 | 72.95 | (69.94, 75.96) | 20.52 | **0.009** | **0.021** |

Abbreviations: CI: confidence interval; SD: standard deviation

^a^ P values are obtained from Wald test without (crude) and with adjustment for age (adjusted for age).

P values <0.05 are indicated in bold.

**Table S8**: SF-36 v2 health domain subscales: mean (p scores) with 95% CI, SD, p values from Wald test (global test) without and with adjustment for age according to children in the household (no, yes).

|  | **No children in household** | | | **Children in household** | | |  |  |
| --- | --- | --- | --- | --- | --- | --- | --- | --- |
| **Scale** | **Mean p score** | **95% CI** | **SD** | **Mean p score** | **95% CI** | **SD** | **p value** | |
|  |  |  |  |  |  |  | **crude** | **adjusted for age** |
| Physical Functioning (PF) | 90.41 | (89.21, 91.61) | 17.93 | 94.56 | (93.11, 96.01) | 11.38 | **<0.001** | **0.046** |
| Physical Role Functioning (RP) | 86.03 | (84.60, 87.46) | 21.27 | 88.51 | (86.32, 90.71) | 16.93 | 0.063 | 0.803 |
| Bodily Pain (BP) | 74.06 | (72.25, 75.87) | 26.57 | 76.02 | (72.86, 79.18) | 24.18 | 0.291 | 0.507 |
| General Health Perceptions (GH) | 75.85 | (74.67, 77.03) | 17.41 | 75.00 | (72.88, 77.13) | 16.07 | 0.498 | 0.068 |
| Vitality (VT) | 63.43 | (62.21, 64.64) | 17.52 | 61.89 | (59.93, 63.85) | 15.48 | 0.190 | 0.829 |
| Social Role Functioning (SF) | 86.21 | (84.83, 87.59) | 20.04 | 84.82 | (82.22, 87.42) | 19.71 | 0.356 | 0.705 |
| Emotional Role Functioning (RE) | 87.84 | (86.51, 89.16) | 19.22 | 87.73 | (85.30, 90.15) | 18.31 | 0.937 | 0.641 |
| Mental Health (MH) | 75.48 | (74.35, 76.61) | 16.41 | 73.53 | (71.58, 75.47) | 14.86 | 0.089 | 0.523 |

Abbreviations: CI: confidence interval; SD: standard deviation

^a^ P values are obtained from Wald test without (crude) and with adjustment for age (adjusted for age).

P values <0.05 are indicated in bold.

**Table S9**: SF-36 v2 health domain subscales: mean (p scores) with 95% CI, SD, p values from Wald test (global test) without and with adjustment for age according to the presence of a chronic condition or a health problem (no, yes).

|  | **No chronic condition or health problem** | | | **Chronic condition or health problem** | | |  |  |
| --- | --- | --- | --- | --- | --- | --- | --- | --- |
| **Scale** | **Mean p score** | **95% CI** | **SD** | **Mean p score** | **95% CI** | **SD** | **p value** | |
|  |  |  |  |  |  |  | **crude** | **adjusted for age** |
| Physical Functioning (PF) | 94.72 | (93.67, 95.76) | 13.21 | 85.69 | (83.87, 87.52) | 20.75 | **<0.001** | **<0.001** |
| Physical Role Functioning (RP) | 91.34 | (90.15, 92.53) | 14.83 | 78.98 | (76.69, 81.26) | 25.74 | **<0.001** | **<0.001** |
| Bodily Pain (BP) | 83.43 | (81.84, 85.02) | 20.54 | 61.29 | (58.68, 63.90) | 27.91 | **<0.001** | **<0.001** |
| General Health Perceptions (GH) | 81.56 | (80.52, 82.60) | 12.99 | 66.67 | (64.92, 68.42) | 19.29 | **<0.001** | **<0.001** |
| Vitality (VT) | 65.85 | (64.56, 67.14) | 16.18 | 59.33 | (57.71, 60.94) | 17.98 | **<0.001** | **<0.001** |
| Social Role Functioning (SF) | 88.62 | (87.25, 89.98) | 16.92 | 81.72 | (79.60, 83.85) | 23.62 | **<0.001** | **<0.001** |
| Emotional Role Functioning (RE) | 89.39 | (88.04, 90.74) | 16.82 | 85.03 | (83.02, 87.05) | 22.34 | **<0.001** | **<0.001** |
| Mental Health (MH) | 76.51 | (75.35, 77.67) | 14.59 | 72.85 | (71.20, 74.50) | 18.17 | **<0.001** | **<0.001** |

Abbreviations: CI: confidence interval; SD: standard deviation

^a^ P values are obtained from Wald test without (crude) and with adjustment for age (adjusted for age).

P values <0.05 are indicated in bold.

**Table S10**: PCS, MCS: mean (T scores) with 95% CI, SD and p values obtained from Wald test (global test) without and with adjustment for age for the whole study sample and subsamples according to sex, age, migration background, educational achievement, employment status, partnership, civil status, children in the household, presence of chronic condition or health problem, and questionnaire language.

|  | **PCS** | | | **p value** | |  | **MCS** | | | **p value ^a^** | |
| --- | --- | --- | --- | --- | --- | --- | --- | --- | --- | --- | --- |
| **Characteristics** | **Mean T score** | **95% CI** | **SD** | **crude** | **adjusted for age** |  | **Mean T score** | **95% CI** | **SD** | **crude** | **adjusted for age** |
| **Whole sample** |  |  |  |  |  |  |  |  |  |  |  |
|  | 50.00 | (49.42, 50.58) | 10.00 |  |  |  | 50.00 | (49.41, 50.59) | 10.00 |  |  |
| **Sex** |  |  |  | **0.012** | **<0.001** |  |  |  |  | **<0.001** | **<0.001** |
| Men | 50.76 | (49.93, 51.59) | 8.83 |  |  |  | 51.40 | (50.62, 52.18) | 8.26 |  |  |
| Women | 49.29 | (48.49, 50.08) | 11.03 |  |  |  | 48.69 | (47.82, 49.57) | 11.44 |  |  |
| **Age** |  |  |  | **<0.001** | - |  |  |  |  | **<0.001** | - |
| 18-25 years | 55.36 | (54.11, 56.61) | 5.67 |  |  |  | 45.11 | (42.63, 47.59) | 10.37 |  |  |
| 26-35 years | 53.41 | (52.08, 54.73) | 7.56 |  |  |  | 47.93 | (46.47, 49.39) | 8.07 |  |  |
| 36-45 years | 53.05 | (52.14, 53.97) | 7.08 |  |  |  | 48.81 | (47.53, 50.10) | 9.34 |  |  |
| 46-55 years | 48.89 | (47.69, 50.10) | 10.00 |  |  |  | 50.48 | (49.29, 51.66) | 10.16 |  |  |
| 56-65 years | 46.49 | (45.01, 47.97) | 11.52 |  |  |  | 51.84 | (50.47, 53.21) | 10.82 |  |  |
| ≥66 years | 44.51 | (43.01, 46.01) | 12.19 |  |  |  | 54.13 | (52.96, 55.30) | 8.97 |  |  |
| **Migration background** |  |  |  | 0.123 | 0.746 |  |  |  |  | **0.002** | **0.016** |
| No | 49.69 | (49.04, 50.34) | 10.72 |  |  |  | 50.68 | (50.04, 51.32) | 10.41 |  |  |
| Yes | 50.74 | (49.57, 51.92) | 8.18 |  |  |  | 48.39 | (47.10, 49.69) | 8.65 |  |  |
| **Education** |  |  |  | **<0.001** | **<0.001** |  |  |  |  | **0.047** | 0.156 |
| Compulsory schooling | 47.57 | (44.93, 50.22) | 12.51 |  |  |  | 46.95 | (44.47, 49.43) | 11.88 |  |  |
| Vocational training | 49.62 | (48.75, 50.49) | 10.24 |  |  |  | 50.23 | (49.32, 51.14) | 10.45 |  |  |
| Upper secondary education | 49.57 | (48.24, 50.90) | 9.89 |  |  |  | 50.97 | (49.64, 52.29) | 9.64 |  |  |
| University education | 52.07 | (51.08, 53.05) | 8.20 |  |  |  | 49.99 | (48.86, 51.11) | 8.66 |  |  |
| **Employment** |  |  |  | **<0.001** | **<0.001** |  |  |  |  | **<0.001** | **0.010** |
| Employed | 51.39 | (50.77, 52.00) | 8.54 |  |  |  | 50.03 | (49.37, 50.69) | 9.06 |  |  |
| Unemployed | 49.13 | (47.04, 51.23) | 12.13 |  |  |  | 45.42 | (43.08, 47.76) | 13.14 |  |  |
| Retired | 44.26 | (42.72, 45.79) | 12.43 |  |  |  | 54.17 | (53.00, 55.34) | 9.05 |  |  |

**Table S10 continued**

|  | **PCS** | | | **p value** | |  | **MCS** | | | **p value ^a^** | |
| --- | --- | --- | --- | --- | --- | --- | --- | --- | --- | --- | --- |
| **Characteristics** | **Mean T score** | **95% CI** | **SD** | **crude** | **adjusted for age** |  | **Mean T score** | **95% CI** | **SD** | **crude** | **adjusted for age** |
| **Partnership** |  |  |  | 0.285 | **0.005** |  |  |  |  | **0.006** | 0.071 |
| Yes | 50.26 | (49.62, 50.90) | 9.64 |  |  |  | 50.57 | (49.91, 51.22) | 9.71 |  |  |
| No | 49.44 | (48.08, 50.81) | 10.86 |  |  |  | 48.37 | (46.95, 49.80) | 10.73 |  |  |
| **Civil status** |  |  |  | **<0.001** | 0.467 |  |  |  |  | **<0.001** | **0.013** |
| Single | 52.95 | (52.03, 53.86) | 8.37 |  |  |  | 48.12 | (46.98, 49.25) | 9.65 |  |  |
| Married | 48.89 | (48.08, 49.70) | 10.00 |  |  |  | 51.43 | (50.65, 52.20) | 9.62 |  |  |
| Divorced or widowed | 47.17 | (45.49, 48.85) | 11.83 |  |  |  | 49.48 | (47.85, 51.11) | 11.26 |  |  |
| **Children in household** |  |  |  | **0.005** | 0.941 |  |  |  |  | **0.030** | 0.719 |
| No | 49.64 | (48.94, 50.34) | 10.47 |  |  |  | 50.36 | (49.65, 51.06) | 10.11 |  |  |
| Yes | 51.44 | (50.39, 52.48) | 7.90 |  |  |  | 48.79 | (47.57, 50.02) | 9.39 |  |  |
| **Chronic condition or health problem** | |  |  | **<0.001** | **<0.001** |  |  |  |  | 0.341 | **0.002** |
| No | 53.59 | (53.04, 54.13) | 6.95 |  |  |  | 50.26 | (49.53, 50.99) | 9.11 |  |  |
| Yes | 44.56 | (43.51, 45.62) | 11.53 |  |  |  | 49.65 | (48.63, 50.67) | 11.29 |  |  |
| **Questionnaire language** |  |  |  | **0.002** | **<0.001** |  |  |  |  | **<0.001** | **<0.001** |
| German | 50.67 | (50.02, 51.32) | 9.83 |  |  |  | 51.14 | (50.51, 51.77) | 9.42 |  |  |
| French | 48.08 | (46.74, 49.41) | 10.34 |  |  |  | 46.48 | (44.97, 47.99) | 11.17 |  |  |
| Italian | 49.07 | (46.40, 51.75) | 9.50 |  |  |  | 49.56 | (47.42, 51.70) | 8.28 |  |  |

Abbreviations: PCS: Physical Component Summary; MCS: Mental Component Summary; CI: confidence interval; SD: standard deviation

^a^ P values are obtained from Wald test (global test) without (crude) and with adjustment for age (adjusted for age).

P values <0.05 are indicated in bold.

**Table S11**: Multivariable regression analyses for Physical Component Summary (PCS) and Mental Component Summary (MCS) for the weighted sample including characteristics being significantly (p<0.05) associated with PCS and MCS, respectively when adjusting for age (see Table S10).

|  |  | **PCS (n=1113, weighted sample)** | | |  | **MCS (n=1136, weighted sample)** | | |
| --- | --- | --- | --- | --- | --- | --- | --- | --- |
| **Characteristics** | | **Coef** | **95% CI** | **p value** |  | **Coef** | **95% CI** | **p value** |
| Sex: women | | -1.02 | (-2.06, 0.02) | 0.055 |  | -1.75 | (-2.90, -0.59) | **0.003** |
| Age [years] | | -0.18 | (-0.23, -0.14) | **<0.001** |  | 0.16 | (0.10,0.22) | **<0.001** |
| Migration background: Yes | | - | - | - |  | -0.80 | (-2.19, 0.58) | 0.255 |
| Education | |  |  | **0.036*** |  |  |  |  |
|  | Compulsory schooling | Reference | - | - |  | - | - | - |
|  | Vocational training | 1.88 | (-0.33, 4.10) | 0.096 |  | - | - | - |
|  | Upper secondary education | 1.86 | (-0.59, 4.32) | 0.137 |  | - | - | - |
|  | University education | 3.09 | (0.77, 5.41) | **0.009** |  | - | - | - |
| Employment | |  |  | 0.173* |  |  |  | **0.035*** |
|  | Unemployed | Reference | - | - |  | Reference | - | - |
|  | Employed | 1.77 | (-0.09, 3.62) | 0.062 |  | 2.99 | (0.65, 5.34) | **0.012** |
|  | Retired | 1.92 | (-0.77, 4.63) | 0.161 |  | 3.64 | (0.65, 6.63) | **0.017** |
| Partnership: Yes | | 0.99 | (-0.30, 2.28) | 0.132 |  | - |  |  |
| Civil status | |  |  |  |  |  |  | 0.054* |
|  | Single | - | - | - |  | Reference | - | - |
|  | Married | - | - | - |  | -0.01 | (-1.54, 1.52) | 0.988 |
|  | Divorced or widowed | - | - | - |  | -2.08 | (-4.20, 0.04) | 0.054 |
| Chronic condition: Yes | | -7.39 | (-8.53, -6.25) | **<0.001** |  | -1.49 | (-2.73, -0.26) | **0.018** |
| Questionnaire language | |  |  | **0.003*** |  |  |  | **<0.001*** |
|  | German | Reference | - | - |  | Reference | - | - |
|  | French | -2.38 | (-3.73, -1.02) | **0.001** |  | -4.20 | (-5.74, -2.66) | **<0.001** |
|  | Italian | -0.82 | (-3.21, 1.57) | 0.502 |  | -0.75 | (-3.22, 1.72) | 0.550 |

Abbreviations: PCS: Physical Component Summary; MCS: Mental Component Summary; Coef, coefficient; CI: confidence interval

* Global p values for categorical variables are obtained from Wald test.

P values <0.05 are indicated in bold.

**Table S12**: Characteristics of study samples weighting coefficients and p scores were based on for Switzerland, USA, Germany, UK, New Zealand, and Australia.

| **Contribution** | **Country** | **SF-36 version** | **Time of data collection** | **Sample size** | **Response rate [%]** | **Age distribution** | **Sex distribution** | **Health status** | **Mode of data collection** |
| --- | --- | --- | --- | --- | --- | --- | --- | --- | --- |
| Coefficients and p scores Switzerland | Switzerland | SF-36 version 2 | 2015-2016 | 1209 | 22.7 | 18-75 years; 7.6% 18-25 years 13.6% 26-35 years 19.1% 36-45 years 23.0% 46-55 years 19.2% 56-65 years 17.5% ≥66 years | 41.9% men | 40.5% with chronic condition or health problem | mail survey |
| Coefficients USA [1] | USA | SF-36 version 1 | 1990 | 2474 | 77.1 | ≥18 years 7.0% 18-24 years 19.2% 25-34 years 20.3% 35-44 years 13.7% 45-54 years 10.9% 55-64 years 17.9% 65-74 years 10.6% ≥75 years 0.4% missing | 42.6% men | 84.4% with hypertension 8.7% with congestive heart failure 21.9% with diabetes type 2 4.3% with recent acute myocardial infarction 20.3% with clinical depression | 80% mail survey and 20% telephone survey |
| P scores USA [2] | USA | SF-36 version 2 | 2009 | 4040 | 66* | ≥18 years; 8.0% 18-24 years 13.1% 25-34 years 16.2% 35-44 years 17.2% 45-54 years 19.1% 55-64 years 18.5% 65-74 years 7.9% ≥75 years | 49.4% men | 37.6% with hypertension 3.4% with congestive heart failure 14.3% with diabetes type 2 1.2% with recent acute myocardial infarction 12.5% with clinical depression | online survey |

**Table S12 continued**

| **Contribution** | **Country** | **SF-36 version** | **Time of data collection** | **Sample size** | **Response rate [%]** | **Age distribution** | **Sex distribution** | **Health status** | **Mode of data collection** |
| --- | --- | --- | --- | --- | --- | --- | --- | --- | --- |
| Coefficients Germany [3-9] | Germany | SF-36 version 1 | 1997-1999 | 7124 | 61.4 | 18-79 years; mean 46.1 years; 3.8% 18-19 years 14.3% 20-29 years 21.8% 30-39 years 18.4% 40-49 years 19.1% 50-59 years 14.5% 60-69 years 8.1% 70-79 years | 51.3% men | 2.5% with myocardial infarction (lifetime prevalence) 1.6% with stroke (lifetime prevalence) 4.7% men and 5.6% women with diabetes (prevalence) 30.0% men and 26.9% women with hypertension 6.3% with affective disorders (12-months prevalence) | written questionnaire in study centers |
| P scores Germany [10, 11] | Germany | SF-36 version 2 | 2008-2011 | 7988 | 64 | 18-79 years; 13.4% 18-29 years 12.7% 30-39 years 19.3% 40-49 years 19.9% 50-59 years 19.3% 60-69 years 15.4% 70-79 years | 47.4% men | *not reported* | written questionnaire in study centers |
| Coefficients and p scores UK [12] | UK | SF-36 version 2 | 1997 | 8889 | 64.4 | 18-64 years | 43.4% men | 36.6% with longstanding illness | mail survey |

**Table S12 continued**

| **Contribution** | **Country** | **SF-36 version** | **Time of data collection** | **Sample size** | **Response rate [%]** | **Age distribution** | **Sex distribution** | **Health status** | **Mode of data collection** |
| --- | --- | --- | --- | --- | --- | --- | --- | --- | --- |
| Coefficients and p scores New Zealand [13-15] | New Zealand | SF-36 version 2 | 2006-2007 | 12488 | 68 | ≥15 years; 4.5% 15-17 years 8.8% 18-24 years 16.7% 25-34 years 20.6% 35-44 years 16.7% 45-54 years 13.8% 55-64 years 10.4% 65-74 years 8.5% ≥75 years | 42.2% men | 65.7% with health condition | face-to-face interviews |
| Coefficients and p scores Australia [16] | Australia | SF-36 version 2 | 2004 | 3015 | 72 | ≥15 years; mean 45.3 years; SD=18.7 years | 49.1% men | *not reported* | face-to-face interviews |

* Combined response rate for the standard and the acute form of the SF-36 version 2; separate response rates not reported.

**Figure S1**: Rotated factor loadings of the SF-36v2 health domain subscales on the two summary measures Physical Component Summary (PCS) and Mental Component Summary (MCS).


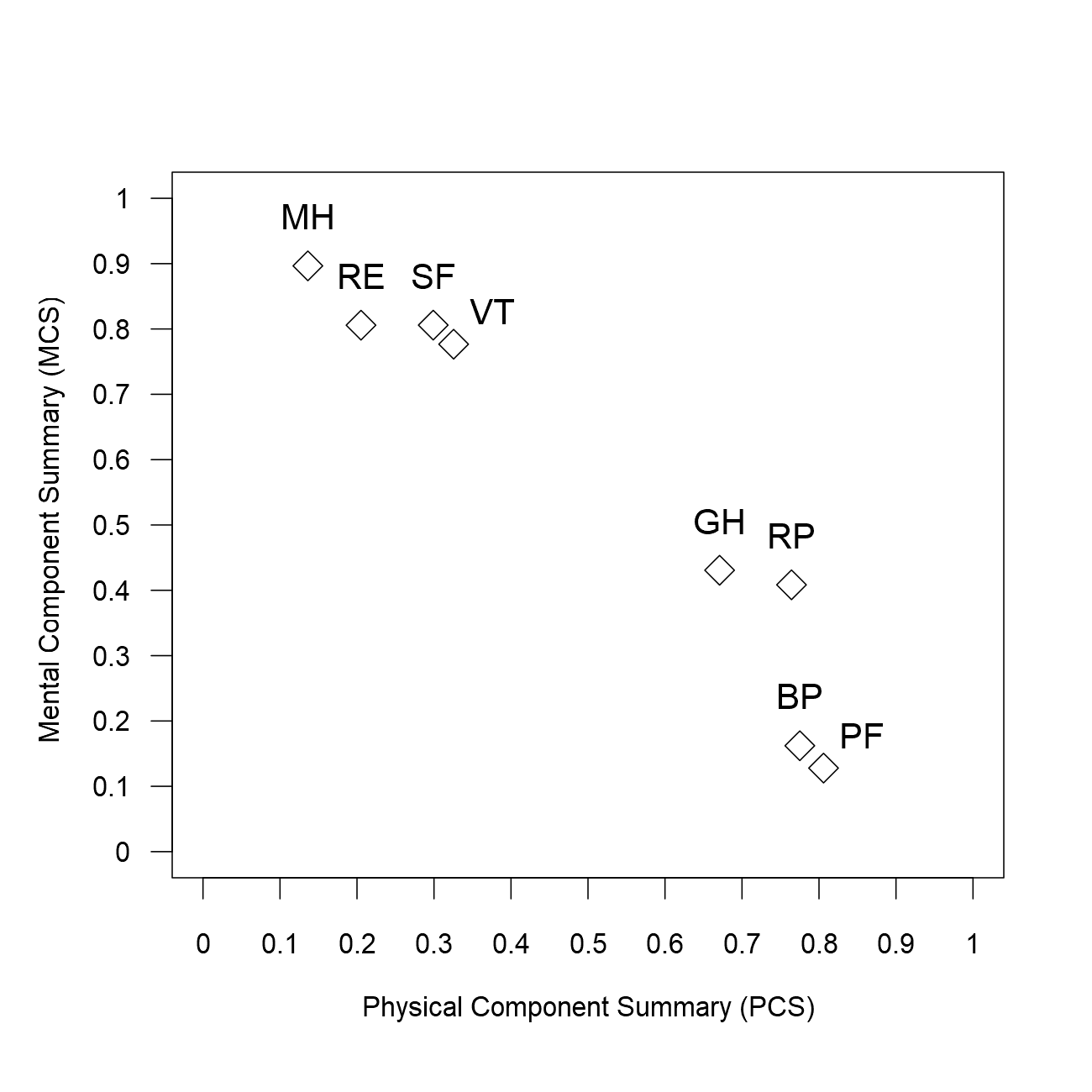


Abbreviations: PCS: Physical Component Summary; MCS: Mental Component Summary; PF: Physical functioning; RP Physical role functioning; BP: Bodily pain; GH: General health perceptions; VT: Vitality; SF: Social role functioning; RE: Emotional role functioning; MH: Mental health

**References**

1. Ware, J.E., Jr., *SF-36 Health Survey Manual & Interpretation Guide*. 1993, The Health Institute, New England Medical Center: Boston, Massachusetts.

2. Maruish, M.E., *User’s Manual for the SF-36v2 Health Survey (3rd ed.)*. 2011, Lincoln, RI: QualityMetric Incorporated.

3. Ellert, U. and B.M. Kurth, *[Methodological views on the SF-36 summary scores based on the adult German population].* Bundesgesundheitsblatt Gesundheitsforschung Gesundheitsschutz, 2004. **47**(11): p. 1027-32.

4. Thamm, M., *[Blood pressure in Germany--current status and trends].* Gesundheitswesen, 1999. **61**: p. S90-3.

5. Thefeld, W., *[Prevalence of diabetes mellitus in the adult German population].* Gesundheitswesen, 1999. **61**: p. S85-9.

6. Thefeld, W., H. Stolzenberg, and B.M. Bellach, *[The Federal Health Survey: response, composition of participants and non-responder analysis].* Gesundheitswesen, 1999. **61**: p. S57-61.

7. Wiesner, G., J. Grimm, and E. Bittner, *[Stroke: prevalence, incidence, trends, East-West comparison. Initial results of the 1998 Federal Health Survey].* Gesundheitswesen, 1999. **61**: p. S79-84.

8. Wiesner, G., J. Grimm, and E. Bittner, *[Incidence of myocardial infarct in Germany: prevalence, incidence trends, East-West comparison].* Gesundheitswesen, 1999. **61**: p. S72-8.

9. Wittchen, H.U., et al., *[Affective, somatoform and anxiety disorders in Germany--initial results of an additional federal survey of "psychiatric disorders"].* Gesundheitswesen, 1999. **61**: p. S216-22.

10. Ellert, U. and B.M. Kurth, *[Health related quality of life in adults in Germany: results of the German Health Interview and Examination Survey for Adults (DEGS1)].* Bundesgesundheitsblatt Gesundheitsforschung Gesundheitsschutz, 2013. **56**(5-6): p. 643-9.

11. Kamtsiuris, P., et al., *[The first wave of the German Health Interview and Examination Survey for Adults (DEGS1): sample design, response, weighting and representativeness].* Bundesgesundheitsblatt Gesundheitsforschung Gesundheitsschutz, 2013. **56**(5-6): p. 620-30.

12. Jenkinson, C., et al., *Assessment of the SF-36 version 2 in the United Kingdom.* J Epidemiol Community Health, 1999. **53**(1): p. 46-50.

13. Frieling, M.A., W.R. Davis, and G. Chiang, *The SF-36v2 and SF-12v2 health surveys in New Zealand: norms, scoring coefficients and cross-country comparisons.* Aust N Z J Public Health, 2013. **37**(1): p. 24-31.

14. Ministry of Health, *Methodology Report for the 2006/07 New Zealand Health Survey*. 2008, Ministry of Health: Wellington.

15. Ministry of Health, *A Portrait of Health. Key Results of the 2006/07 New Zealand Health Survey*. 2008, Ministry of Health: Wellington.

16. Hawthorne, G., et al., *The SF36 Version 2: critical analyses of population weights, scoring algorithms and population norms.* Qual Life Res, 2007. **16**(4): p. 661-73.
