# Supplementary material for: Health-related quality of life in Switzerland: normative data for the SF-36v2 questionnaire
Source: Qual Life Res. 2019 Mar 8;28(7):1963–77. doi: 10.1007/s11136-019-02161-5 (PMC6571102; doi:10.1007/s11136-019-02161-5)
Supplement: Supplementary file 2 — Supplementary material 2 (DOCX 23 KB) [file 11136_2019_2161_MOESM2_ESM.docx]

**Request Data: data that can be requested from the authors**

**Weighted data**

**Table A1**: Item description: mean, standard deviation, number of levels, and imputed values of the items of the SF-36v2 questionnaire based on weighted data.

**Table A2**: Item-subscale correlations (item-rest correlations for the subscales and their respective items; indicated with an asterisk) between the items and the health domain subscales of the SF-36v2 questionnaire based on weighted data.

**Table A3**: Differences between item-subscale correlations and item-rest correlations for the items of the SF-36v2 questionnaire based on weighted data.

**Table A4**: Pearson correlations between the health domain subscales and the summary measures of the SF-36v2 questionnaire; Cronbach’s alpha of the subscales in brackets based on weighted data.

**Non-weighted data**

**Table A5**: Item description: mean, standard deviation, number of levels, and imputed values of the items of the SF-36v2 questionnaire based on non-weighted data.

**Table A6**: Item-subscale correlations (item-rest correlations for the subscales and their respective items; indicated with an asterisk) between the items and the health domain subscales of the SF-36v2 questionnaire based on non-weighted data.

**Table A7**: Differences between item-subscale correlations and item-rest correlations for the items of the SF-36v2 questionnaire based on non-weighted data.

**Table A8**: Pearson correlations between the health domain subscales and the summary measures of the SF-36v2 questionnaire; Cronbach’s alpha of the subscales in brackets based on non-weighted data.

**Table A9**: Spearman correlations between the health domain subscales and the summary measures of the SF-36v2 questionnaire; Cronbach’s alpha of the subscales in brackets based on non-weighted data.
